# Combined Anteversion Technique for Total Hip Arthroplasty With Handheld Accelerometer-Based Navigation System

**DOI:** 10.1016/j.artd.2023.101193

**Published:** 2023-09-18

**Authors:** Diego Alarcon Perico, Christopher N. Warne, Sheng-Hsun Lee, Heather J. Roberts, Rafael J. Sierra

**Affiliations:** Department of Orthopedic Surgery, Mayo Clinic, Rochester, MN, USA

**Keywords:** Navigation, Total hip arthroplasty, Combined version

## Abstract

Aiming for a combined cup and stem anteversion within a target range is one way to assess appropriate prosthetic component orientation and restoration of functional range of motion. We describe a surgical technique that allows the surgeon to assess the combined anteversion using a handheld accelerometer-based navigation system for total hip arthroplasty through a posterior approach. The femur is prepared first, at which time the femoral version is estimated by the surgeon. The acetabular component is then positioned using the navigation system to estimate anteversion, with the goal of providing a combined version of 37° ± 7°. The described technique allows surgeons to achieve the desired intraoperative combined anteversion.

**Level of evidence:**

IV (technical note).

## Introduction

Proper positioning of the acetabular and femoral components during total hip arthroplasty (THA) is crucial to restore anatomic relationships and biomechanical function of the hip [1,2]. Accurate femoral and acetabular anteversion optimize impingement-free functional range of motion and are associated with decreased implant dislocation [3].

The combined anteversion technique has been described previously [4]. This concept was introduced by Ranawat et al. [5] in 1991 and suggests that the ideal sum of anteversion of the cup and the femoral stem should be approximately 45° in women and between 20° and 30° in men. After subsequent research with mathematical and risk calculations [6], it has been recommended that the cup inclination be between 40° and 45° and the combined cup and stem anteversion be determined by the formula: cup anteversion + (0.7 × stem anteversion) = 37° ± 7°. This recommendation of combined anteversion has been adopted clinically and has been correlated with a reduced risk of impingement and hip dislocation [7].

There has been increasing interest in the use of intraoperative navigation and robotics to achieve accurate implant positioning. Specifically, handheld accelerometer-based navigation has been used for total knee arthroplasty, but there is less data supporting its role for THA. Previous papers have shown its accuracy in determining both acetabular component position and leg lengths [8,9]. Placing the acetabular component in the Lewinnek safe zone of 45° of abduction and 15° of anteversion does not seem to be appropriate for all patients. Previous studies have shown that there are still a high number of patients that can dislocate within the acetabular safe zone, suggesting contributions from the femoral component and spine [10]. This is especially important in young patients undergoing THA where a structural deformity has led to osteoarthritis, such as hip dysplasia, impingement, or osteonecrosis [[11], [12], [13], [14]].

We therefore consider that at the time of THA, the hip should be “balanced” with a combined anteversion technique as described previously, where contributions of both the acetabular and femoral sides are considered. The purpose of this paper is therefore to report a technique for combined anteversion using the HipAlign navigation system.

## Surgical technique

**Figure 1:**
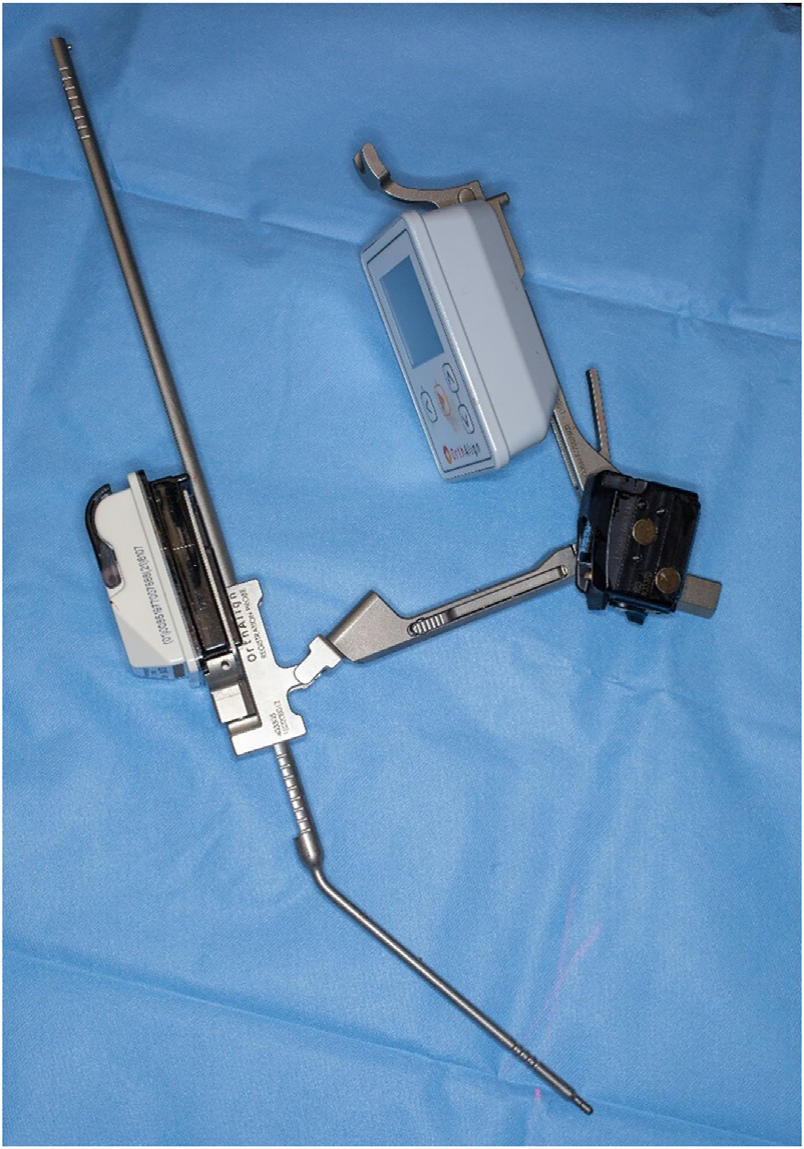
The navigation unit and reference sensor each contain triaxial accelerometers and gyroscopes that communicate wirelessly with one another.

**Figure 2:**
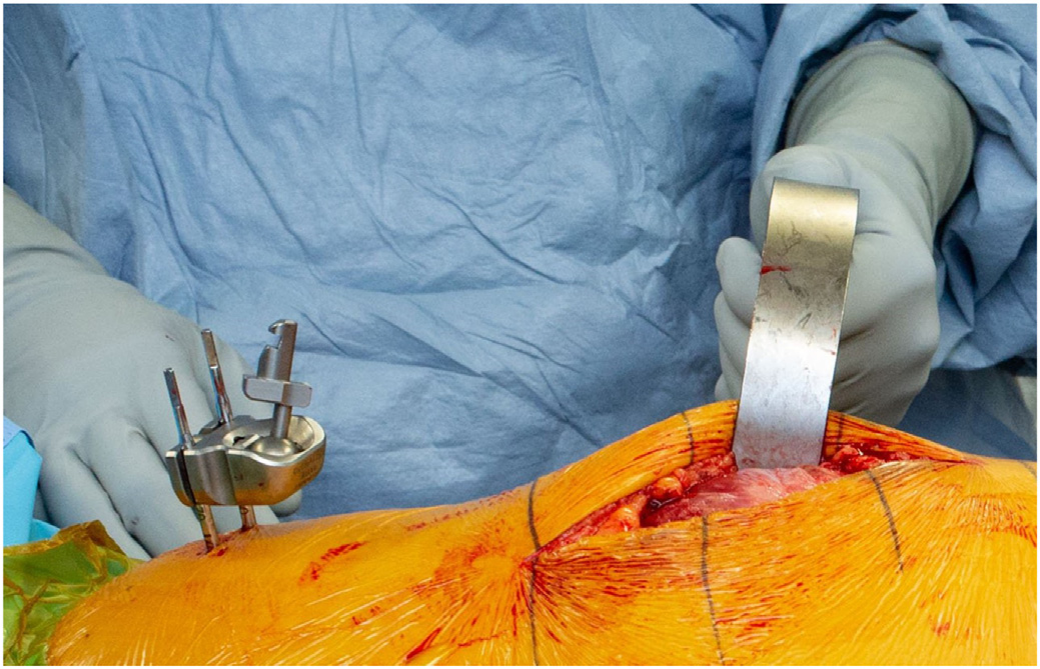
The metal pelvic base and navigation unit were percutaneously secured with 2 pins to the ipsilateral iliac crest using sterile technique.

**Figure 3:**
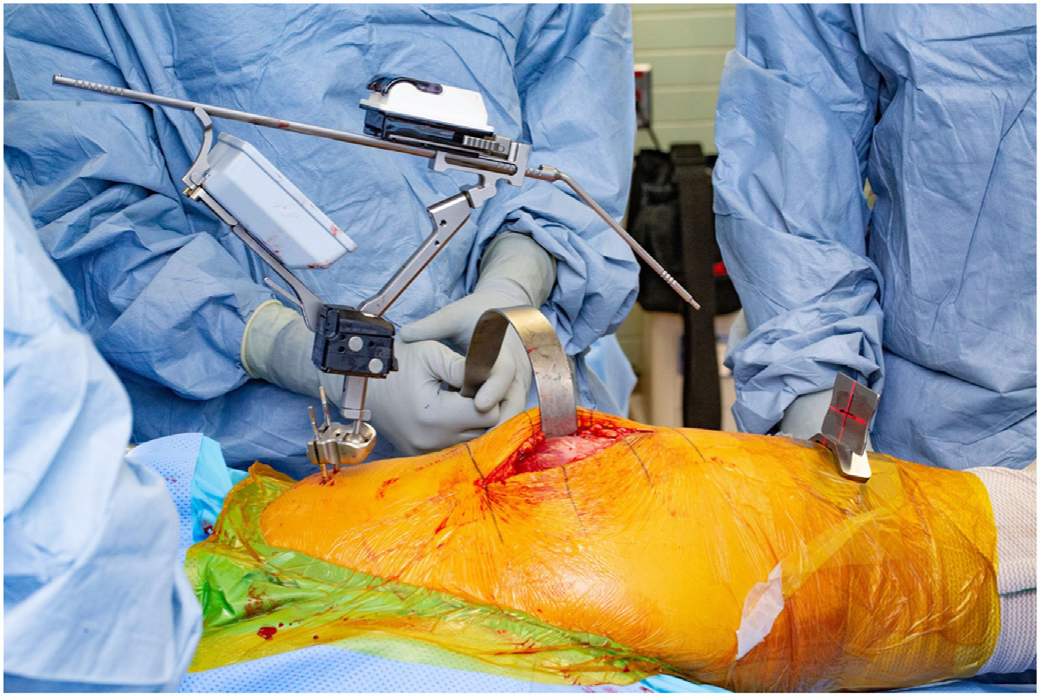
The longitudinal coronal plane of the body was registered by holding the registration probe parallel to the long axis of the body.

In all patients, digital preoperative anteroposterior and lateral radiographs of the hips are performed for preoperative planning with THA templates. Patients receive a THA through a posterolateral approach using a handheld accelerometer-based navigation system (OrthAlign, Aliso Viejo, CA). This system, called HipAlign System (OrthAlign, Aliso Viejo, CA), consists of a disposable navigation unit with a display console and a reference sensor. The navigation unit and reference sensor each contain triaxial accelerometers and gyroscopes that communicate wirelessly with one another (Fig. 1). The navigation and a reference sensor are paired and calibrated on a flat table prior to surgery. The metal pelvic base and navigation unit are percutaneously secured with 2 pins to the ipsilateral iliac crest using sterile technique (Fig. 2). The incision is then made, and the dissection is carried through the subcutaneous tissue and fascia lata. A tack is placed into the greater trochanter to serve as a reference point for the leg length measurement probe. Registration occurs prior to dislocation of the hip. The longitudinal coronal plane of the body is registered by holding the registration probe parallel to the long axis of the body (Fig. 3). For intraoperative leg length measurement, the laser module is attached to the navigation unit, and the thigh plate is positioned laterally on the distal thigh and secured with an adhesive band. Once the initial leg lengths are measured, the hip replacement is carried out in the usual fashion.

**Figure 4:**
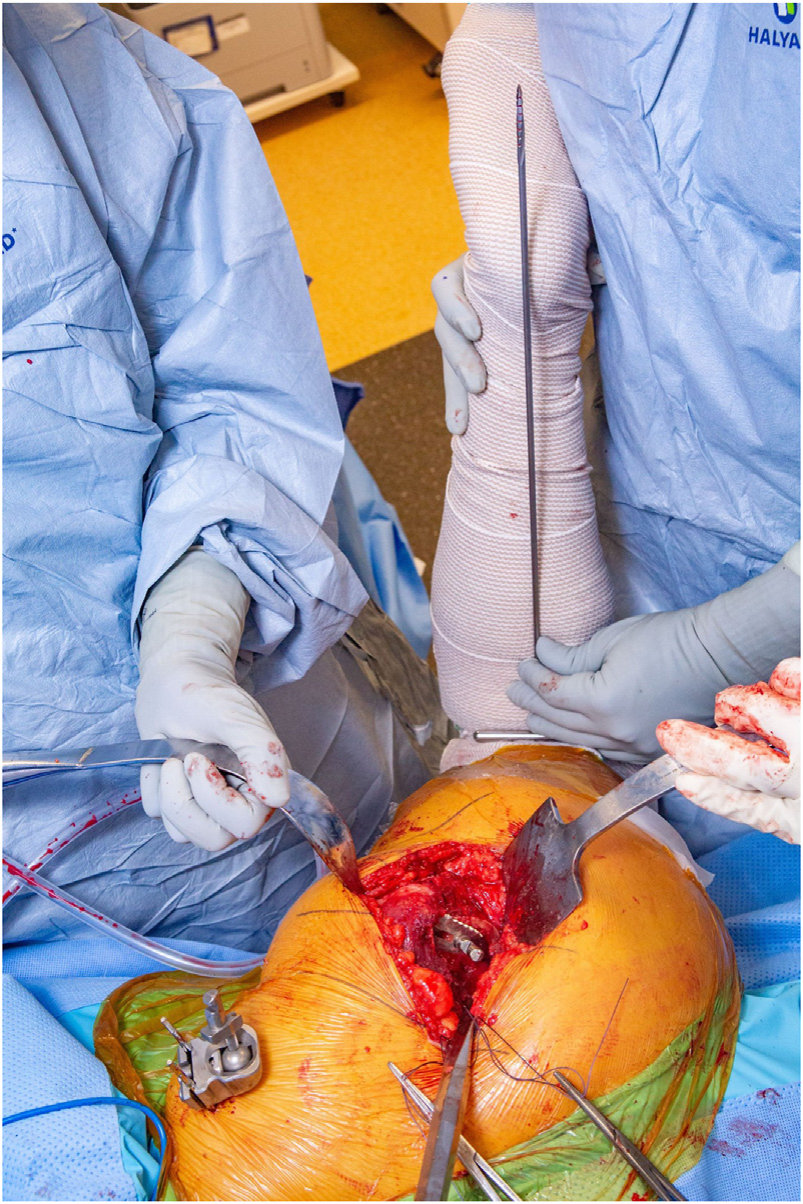
The femoral version is estimated by holding the knee flexed with the tibia in a vertical position. The version is then registered.

After dislocation, the femoral neck cut is made based on the preoperative templating. The native femoral version is then estimated by holding the knee flexed with the tibia in a vertical position. The femur is prepared first, aiming for between 10° and 15° of femoral component anteversion (Fig. 4). Standard primary hip implants that are designed for proximal fixation allow a change in femoral version of approximately 10 degrees from native, and patients with extreme versional abnormalities may require a modular or a cemented stem to correct abnormal femoral version. The femoral component anteversion is then estimated and recorded using the same technique for estimating native femoral version. The broach is left in place during acetabular preparation.

**Figure 5:**
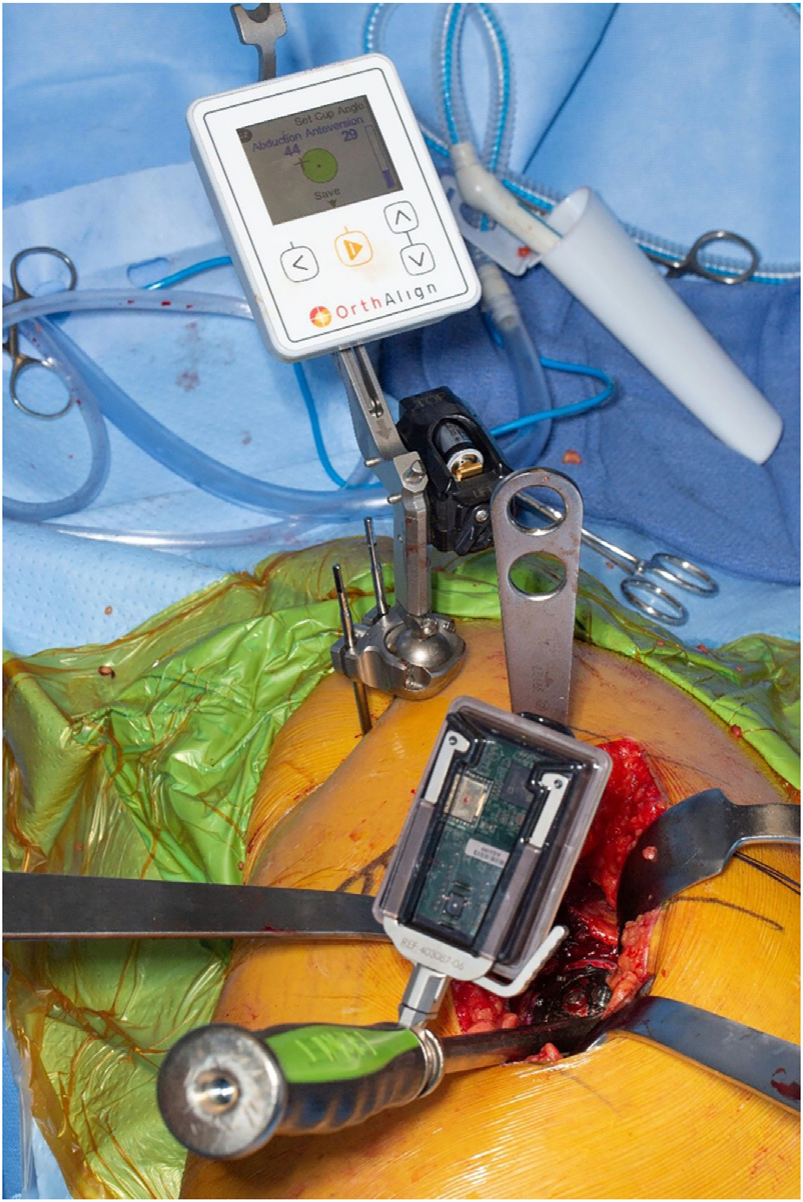
The acetabular version is measured using the navigation system.

The acetabulum is then exposed, the labrum is resected, and the acetabular cavity is sequentially reamed to 1 mm less than the desired acetabular component so that a press-fit can be achieved. The native acetabular version based on the transverse acetabular ligament is then measured using the navigation system (Fig. 5). The native acetabular version will serve as a “double check” for acetabular component version, using the transverse ligament as a reference. Both abduction and anteversion are displayed on the display consul and are used to guide positioning of the cup during implantation. Our target for acetabular abduction is usually 40°; however, in patients with mild hip dysplasia, a slightly more abducted position is acceptable in order to increase acetabular component coverage by native bone. The acetabular anteversion depends on the femoral component version to obtain a combined anteversion goal of 37° ± 7°. The acetabular component is then positioned to achieve the combined anteversion based on femoral version. For example, if the femoral version is 10°, its contribution to combined anteversion is 7° based on the previously described mathematical formula. The acetabular version target would then be 30° in order to obtain a combined version of 37°. If, on the other hand, the femoral version is 20°, then 14° is the contribution from the femoral side, and, therefore, target acetabular version is 25°. The target acetabular version will therefore vary based on position of the femoral component. Care is taken to ensure that the anterior rim of the acetabular component is within the native bone in order to avoid iliopsoas impingement.

Once the implants have been placed, the hip is brought through a range of motion. If the offset and leg length have been restored, the hip should be stable to at least 60° of internal rotation at 90° of flexion, and there should be no impingement of the neck against the posterior acetabular rim in extension and external rotation. If impingement-free range of motion is inadequate at the time of trialing, the first step is to determine whether offset or leg lengths have been restored. Once impingement-free range of motion is deemed appropriate, the acetabular liner is impacted into place, typically a 36 mm polyethylene liner. The broach is then removed, and the final femoral component is impacted into place. The hip is then reduced with a trial head. Leg lengths are measured, and if deemed appropriate, the final femoral head is impacted onto the trunnion.

## Discussion

The use of the HipAlign system for hip arthroplasty has been described previously. Tanino et al. described the technique and demonstrated reliable implant position with the system. The authors reported significantly lower deviations of target cup position within the Lewinnek safe zone with the navigation system [9]. The system has also been shown to aid in accurate implant positioning when performing THA through a direct anterior approach [8].

Femoral version and offset restoration are important contributors to implant stability. This is especially important in patients with early degenerative joint disease, where subtle underlying structural abnormalities have led to early joint degeneration. Patients presenting with arthritis from osteonecrosis, dysplasia, or femoroacetabular impingement have underlying structural deformities of the hip and may have either increased or decreased femoral or acetabular anteversion. It is therefore paramount to use a surgical technique that corrects for these preoperative anatomic abnormalities to optimize impingement-free range of motion. The technique described by Ranawat and Dorr optimizes impingement-free range of motion with a combined version between 25° and 50° and is applicable to the majority of THAs performed as long as offset and leg lengths are restored.

The combined anteversion technique optimizes an impingement-free range of motion. Once the implants are within the combined anteversion safe zone, the hip should be carried through a range of motion and tested in 90° of flexion and internal rotation and in extension and external rotation. If combined anteversion within the target range has been achieved and the patient is not stable to at least 50° of internal rotation at 90° of flexion, then other factors contributing to the decreased range of motion or impingement should be assessed (leg lengths, offset restoration, and bony or soft tissue impingement). By achieving a target combined anteversion with this technique, the surgeon decreases the risk of prosthetic implant position as the cause of impingement.

Previous studies have reported a decrease in dislocation rates with the use of navigation systems [15,16]. Further evaluation of the technology is needed to determine any potential functional improvements. This navigation system does not account for the contributions that the spine may have on functional range of motion of the hip. Future systems incorporating preoperative spine assessment may aid in decision-making for patients with complex spine diseases that have been associated with a higher risk of dislocation [17].

We describe the technique of combined anteversion for THAs performed through a posterior approach with a handheld accelerometer-based navigation system. The system allows achieving an intraoperative combined anteversion of 37° ± 7°. The proposed technique could lead to decrease risk of dislocation by supporting functional range of motion and minimizing bony and implant impingement.

## Summary

The described technique of combined anteversion in THA with a handheld accelerometer-based navigation system allows surgeons to achieve the desired intraoperative combined anteversion.

## Conflicts of interest

R. Sierra receives royalties from the OrthAlign surgeon council, Zimmer, and Link Orthopaedics; is a paid consultant for Biomet, Link Orthopaedics, OrthoAlign, and Think; receives stock options from OrthAlign; receives research support from Zimmer, Cytori, DePuy, A Johnson & Johnson Company, Orthalign, Stryker, Biomet; receives publishing royalties, financial or material support from Springer; is an editorial/governing board member of the Journal of Arthroplasty; is a board/committee member of the American Association of Hip and Knee Surgeons, Anchor study group, Knee Society, and Muller Foundation. All other authors declare no potential conflicts of interest.

For full disclosure statements refer to https://doi.org/10.1016/j.artd.2023.101193.

## Appendix A Supplementary data

Conflict of Interest Statement for Diego

Conflict of Interest Statement for Lee

Conflict of Interest Statement for Sierra

Conflict of Interest Statement for Roberts

Conflict of Interest Statement for Warne

## References

[1] D'Lima D.D., Urquhart A.G., Buehler K.O., Walker R.H., Colwell C.W. (2000). The effect of the orientation of the acetabular and femoral components on the range of motion of the hip at different head-neck ratios. J Bone Joint Surg Am.

[2] Daines B.K., Dennis D.A. (2012). The importance of acetabular component position in total hip arthroplasty. Orthop Clin North Am.

[3] Seagrave K.G., Troelsen A., Malchau H., Husted H., Gromov K. (2017). Acetabular cup position and risk of dislocation in primary total hip arthroplasty. Acta Orthop.

[4] Amuwa C., Dorr L.D. (2008). The combined anteversion technique for acetabular component anteversion. J Arthroplasty.

[5] Ranawac C.S., Maynard M.J. (1991). Modern technique of cemented total hip arthroplasty. Tech Orthop.

[6] Widmer K.H., Zurfluh B. (2004). Compliant positioning of total hip components for optimal range of motion. J Orthop Res.

[7] Nakashima Y., Hirata M., Akiyama M., Itokawa T., Yamamoto T., Motomura G. (2014). Combined anteversion technique reduced the dislocation in cementless total hip arthroplasty. Int Orthop.

[8] Tanino H., Nishida Y., Mitsutake R., Ito H. (2021). Accuracy of a portable accelerometer-based navigation system for cup placement and intraoperative leg length measurement in total hip arthroplasty: a cross-sectional study. BMC Musculoskelet Disord.

[9] Tanino H., Nishida Y., Mitsutake R., Ito H. (2020). Portable accelerometer-based navigation system for cup placement of total hip arthroplasty: a prospective, randomized, controlled study. J Arthroplasty.

[10] Abdel M.P., von Roth P., Jennings M.T., Hanssen A.D., Pagnano M.W. (2016). What safe zone? The vast majority of dislocated THAs are within the Lewinnek safe zone for acetabular component position. Clin Orthop Relat Res.

[11] Wyles C.C., Heidenreich M.J., Jeng J., Larson D.R., Trousdale R.T., Sierra R.J. (2017). The John Charnley award: redefining the natural history of osteoarthritis in patients with hip dysplasia and impingement. Clin Orthop Relat Res.

[12] Wyles C.C., Vargas J.S., Heidenreich M.J., Mara K.C., Peters C.L., Clohisy J.C. (2019). Natural history of the dysplastic hip following modern periacetabular osteotomy. J Bone Joint Surg Am.

[13] Ollivier M., Lunebourg A., Abdel M.P., Parratte S., Argenson J.N. (2016). Anatomical findings in patients undergoing total hip arthroplasty for idiopathic femoral head osteonecrosis. J Bone Joint Surg Am.

[14] Leunig M., Beaulé P.E., Ganz R. (2009). The concept of femoroacetabular impingement: current status and future perspectives. Clin Orthop Relat Res.

[15] Bohl D.D., Nolte M.T., Ong K., Lau E., Calkins T.E., Della Valle C.J. (2019). Computer-assisted navigation is associated with reductions in the rates of dislocation and acetabular component revision following primary total hip arthroplasty. J Bone Joint Surg Am.

[16] Agarwal S., Eckhard L., Walter W.L., Peng A., Hatton A., Donnelly B. (2021). The use of computer navigation in total hip arthroplasty is associated with a reduced rate of revision for dislocation: a study of 6,912 navigated THA procedures from the Australian Orthopaedic Association National Joint Replacement Registry. J Bone Joint Surg Am.

[17] Vigdorchik J., Eftekhary N., Elbuluk A., Abdel M.P., Buckland A.J., Schwarzkopf R.S. (2019). Evaluation of the spine is critical in the workup of recurrent instability after total hip arthroplasty. Bone Joint Lett J.

