# Supplementary material for: Combined Anteversion Technique for Total Hip Arthroplasty With Handheld Accelerometer-Based Navigation System
Source: Arthroplast Today. 2023 Sep 18;23:101193. doi: 10.1016/j.artd.2023.101193 (PMC10514434; doi:10.1016/j.artd.2023.101193)
Supplement: Conflict of Interest Statement for Sierra [file mmc3.pdf]

CONFLICT OF INTEREST STATEMENT

American Association of Hip and Knee Surgeons

(Adopted from the American Academy of Orthopaedic Surgeons disclosure statement)

The following form **must be filled out completely and submitted by each author (example, 6 authors, 6 forms). All items require a response. If there is no relevant disclosure for a given item, enter "*None.*"**

Combined Version Technique for Total Hip Arthroplasty with Handheld Accelerometer-Based Navigation System

---

Manuscript Title

1. Royalties from a company or supplier (The following conflicts were disclosed)

Orthalign: IP royalties

2. Speakers bureau/paid presentations for a company or supplier (The following conflicts were disclosed)

Biomet: Paid consultant; Paid presenter or speaker

3A. Paid employee for a company or supplier (The following conflicts were disclosed)

Zimmer: IP royalties
Biomet: Paid consultant
Link Orthopaedics: IP royalties; Paid consultant
Orthoalign: Paid consultant
T hink: Paid consultant

3B. Paid consultant for a company or supplier (The following conflicts were disclosed)

3C. Unpaid consultants for a company or supplier (The following conflicts were disclosed)

4. Stock or stock options in a company or supplier (The following conflicts were disclosed)

Orthoalign Stock or stock Options

5. Research support from a company or supplier as a Principal Investigator (The following conflicts were disclosed)

Zimmer: Research support Cytori: Research support
DePuy, A Johnson & Johnson Company: Research support
Orthalign, Research support
Stryker, Biomet: Research support

6. Other financial or material support from a company or supplier (The following conflicts were disclosed)

7. Royalties, financial or material support from publishers (The following conflicts were disclosed)

Springer: Publishing royalties, financial or material support

8. Medical/Orthopaedic publications editorial/governing board (The following conflicts were disclosed)

9. Board member/committee appointments for a society (The following conflicts were disclosed)

American Association of Hip and Knee Surgeons: Board or committee member
Anchor study group: Board or committee member
Journal of Arthroplasty: Editorial or governing board
Knee Society: Board or committee member
Muller Foundation: Board or committee member

Each author must sign AND print or type his/her name, date and submit a separate form

In addition, one BLINDED Conflict of Interest form (no author names used) should be submitted per manuscript with all author disclosures.

Rafael Jose Sierra, MD, FAAOS	Rafael J. Sierra, MD Digitally signed by Rafael J. Sierra, MD Date: 2023.03.17 11:28:28 -05'00'	
Author Name (Print or Type)	Author Signature	Date
